# Protein Synthesis Inhibition and Activation of the c-Jun N-Terminal Kinase Are Potential Contributors to Cisplatin Ototoxicity

**DOI:** 10.3389/fncel.2017.00303

**Published:** 2017-09-27

**Authors:** Brian D. Nicholas, Shimon Francis, Elizabeth L. Wagner, Sibo Zhang, Jung-Bum Shin

**Affiliations:** Department of Neuroscience, University of Virginia, Charlottesville, VA, United States

**Keywords:** Cisplatin, ototoxicity, inner ear, hair cell, protein synthesis, BONCAT, JNK, mTOR

## Abstract

Cisplatin has been regarded as an effective and versatile chemotherapeutic agent for nearly 40 years. Though the associated dose-dependent ototoxicity is known, the cellular mechanisms by which cochleovestibular hair cell death occur are not well understood. We have previously shown that aminoglycoside ototoxicity is mediated in part by cytosolic protein synthesis inhibition. Despite a lack of molecular similarity, aminoglycosides were shown to elicit similar stress pathways to cisplatin. We therefore reasoned that there may be some role of protein synthesis inhibition in cisplatin ototoxicity. Employing a modification of the bioorthogonal noncanonical amino acid tagging (BONCAT) method, we evaluated the effects of cisplatin on cellular protein synthesis. We show that cisplatin inhibits cellular protein synthesis in organ of Corti explant cultures. Similar to what was found after gentamicin exposure, cisplatin activates both the c-Jun N-terminal kinase (JNK) and mammalian target of rapamycin (mTOR) pathways. In contrast to aminoglycosides, cisplatin also inhibits protein synthesis in all cochlear cell types. We further demonstrate that the multikinase inhibitor sorafenib completely prevents JNK activation, while providing only moderate hair cell protection. Simultaneous stimulation of cellular protein synthesis by insulin, however, significantly improved hair cell survival in culture. The presented data provides evidence for a potential role of protein synthesis inhibition in cisplatin-mediated ototoxicity.

## Introduction

Cisplatin is a long-established therapeutic agent for a variety of malignant diseases (Rybak, 2007; Langer et al., 2013; Landier, 2016). It is associated with dose-dependent nephro- and ototoxicity, though the cellular mechanisms by which these processes occur are poorly understood (Humes, 1999; Karasawa and Steyger, 2015). Within the cochlea, cisplatin uptake results in the degradation of outer hair cells with subsequent loss of inner hair cells and supporting cells within the organ of Corti at higher doses (Hawkins, 1976). A similar loss of sensory hair cells within the vestibular end organs has been noted after exposure to cisplatin (Black et al., 1982; Zhang et al., 2003; Baker et al., 2015). The incidence of ototoxicity among those receiving cisplatin ranges from 35% to 100% (Benedetti Panici et al., 1993; Nitz et al., 2013; Malgonde et al., 2015). Reflecting the incomplete understanding of the cellular mechanisms underlying cisplatin ototoxicity, clinical trials employing empiric strategies for otoprotection have resulted in mostly mixed results (Marina et al., 2005; Gurney et al., 2014; Yoo et al., 2014).

Protein synthesis and cellular degradation are tightly regulated processes that allow for cells to adapt to a range of environmental conditions. A universal stress response within the cell has been described and nutritional, chemical and ischemic stresses to the cell can alter intracellular protein homeostasis (Gebauer and Hentze, 2004; Sonenberg and Hinnebusch, 2009). In addition to serving as a response to stress conditions, the regulation of protein synthesis and proteolysis, itself, can alter the viability of the cell. An arrest of translation or disruption of protein degradation has been shown to result in neurodegenerative and ototoxic disease processes (Keller, 2006; Kim et al., 2017). The relationship between protein synthesis inhibition and apoptosis, however, is complex. While known inhibitors of protein synthesis such as ricin and anisomycin have been shown to induce apoptosis (Kageyama et al., 2002; Croons et al., 2009), the apoptotic process itself is dependent on cellular protein synthesis (Lockshin and Zakeri, 1992; Mesner et al., 1992). In this way, translational arrest can either promote or inhibit programmed cell death (Rehen et al., 1996), depending on how the balance of pro-survival and pro-death factors is influenced by protein synthesis inhibition in that particular cell type or tissue.

We have previously shown that gentamicin-induced ototoxicity is associated with cellular protein synthesis inhibition (Francis et al., 2013). This is correlated with activation of both the c-Jun N-terminal kinase (JNK) and mammalian target of rapamycin (mTOR) pathways (Francis et al., 2013). There appears to be significant similarity between cellular processes associated with aminoglycoside and cisplatin ototoxity (Schacht et al., 2012). The goal of the present study, then, was to determine if cisplatin ototoxicity is associated with an inhibition of cellular protein synthesis. In addition, we sought to test whether modulation of correlated cell signaling events such as the JNK and mTOR pathways, and/or stimulation of protein synthesis might mitigate cisplatin-induced sensory hair cell loss.

In order to test our hypothesis, we utilized the biorthogonal non-canonical amino acid tagging (BONCAT) method (Dieterich et al., 2007), in which the incorporation of methionine analogs into newly formed proteins serves as a measure for overall cellular protein synthesis activity. We recently modified this method to allow for a cell-by-cell analysis of protein synthesis (Francis et al., 2013). The effects of varying concentrations of cisplatin on cellular protein synthesis in organ of Corti explant cultures were detailed. As seen previously with gentamicin, cisplatin inhibits cellular protein synthesis. We further demonstrate an associated activation of the JNK and mTOR pathways after exposure to cisplatin, which in turn is prevented by the multikinase inhibitor sorafenib. While sorafenib alone only moderately improves hair cell survival, combination with insulin, employed here for its ability to stimulate cellular protein synthesis, significantly improves hair cell survival after cisplatin exposure.

## Materials and Methods

### Animal Care and Handling

This study involves the use of mice. The protocol for care and use of animals was approved by the University of Virginia Animal Care and Use Committee. The University of Virginia is accredited by the American Association for the Accreditation of Laboratory Animal Care. All mouse experiments were performed using the CBA/J inbred mouse strain. Neonatal mouse pups (postnatal day 3 (P3)–P4) were killed by rapid decapitation, and mature mice were killed by CO_2_ asphyxiation followed by cervical dislocation.

### Organotypic Explant Cultures

Mouse cochleae and utricles were dissected in Hank’s balanced salt solution (HBSS, Invitrogen, MA, USA) containing 25 mM HEPES, pH 7.5. The organ of Corti was separated from the spiral lamina and the spiral ligament using fine forceps and attached to the bottom of sterile 35 mm Petri dishes (BD Falcon, NY), with the hair bundle side facing up. The dissection medium was then replaced by two exchanges with culture medium (complete high-glucose DMEM containing 1% FBS, supplemented with ampicillin and ciprofloxacin). Prior to experimental manipulation, explants were pre-cultured for 24 h, to allow acclimatization to the culture conditions (Francis et al., 2013). Cisplatin (TEVA, MD NDC 0703-5748-11, injectable solution, 1 mg/ml) and gentamicin (Sigma, St. Louis, MO, USA) were dissolved in water. Sorafenib and rapamycin (Selleckchem, TX, USA) were dissolved in DMSO (1 mM stock solutions). The organ of Corti was cultured as a whole. Number of experiments (*n*) for quantification of hair cell numbers, activated caspase-3 positive cells and Azidohomoalanine (AHA) uptake indicates number of organs.

### BONCAT and Click-Chemistry Reaction

AHA, incorporated into new proteins, was conjugated via click-chemistry reaction with a biotin moiety, which in turn was detected using streptavidin (SA)-horseradish peroxidase (HRP) for immunoblots and SA-fluorophore for fluorescence microscopy. In contrast to a more complex protocol used in a previous study (Francis et al., 2013), we here used a copper-free version of the click-chemistry reaction (strain-promoted click chemistry; Agard et al., 2004). Here, the azide group on AHA is reacted with a dibenzylcyclooctyne (DBCO)-conjugated biotin (instead of alkyne-biotin) in aqueous solution that does not require copper catalysis. Organotypic explants were cultured for various times in methionine-free medium containing AHA (Invitrogen, catalog #C10102, or Anaspec, Fremont, CA, USA) at a final concentration of 400 μM. After the desired culture time, organs were washed in HBSS for 15 min at 37°C to remove unincorporated AHA. For immunoblots, protein lysates were prepared (10 mg/ml) in protein extraction buffer, and incubated in 50 mM Tris/HCl, pH 7.5, containing 10 μM sulfo-DBCO-biotin conjugate (Click Chemistry Tools, AZ, USA; catalog #A115-10) for 1 h. Proteins were precipitated using methanol/chloroform protein extraction. The protein pellet was resolubilized into 100 μl Laemmli buffer, and 10 μl was run on the gels. For copper-free click reaction in fixed whole-mount organs to be imaged by fluorescence microscopy, explants that have incorporated AHA were fixed, permeabilized and washed in 50 mM Tris/HCl, pH 7.5. Organs were then incubated in 50 mM Tris/HCl, pH 7.5, 0.2% saponin, containing 10 μM sulfo-DBCO-biotin conjugate (Click Chemistry Tools, catalog #A115-10) for 1 h and washed three times in PBS. Biotin was then detected using fluorophore-conjugated SA.

### Quantification of AHA Signal

**Figure 1:**
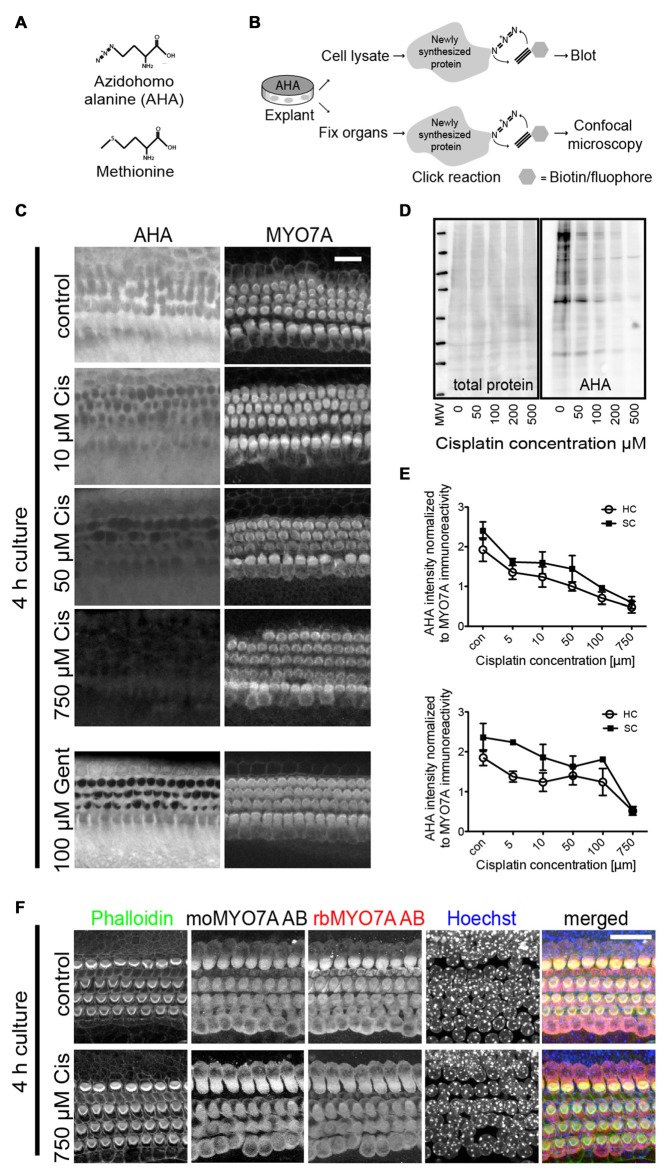
Bioorthogonal noncanonical amino acid tagging (BONCAT) to study protein synthesis within sensory hair cells of mouse explant cultures. **(A)** Chemical structure of methionine and its utilized analog, azidohomoalanine (AHA). **(B)** Schematic of the BONCAT technique using either cell lysates for immunoblot or fixed organs for fluorescence microscopy. **(C)** AHA-biotin immunoreactivity demonstrates dose-dependent inhibition of protein synthesis on a cell-by-cell basis after treatment with cisplatin. P3–4 organ of Corti explants were cultured in growth medium containing AHA, in the presence of varying cisplatin concentrations. After 4 h, prior to the onset of cisplatin-induced cell death or changes in Myosin 7a (MYO7A) levels, explants were fixed and processed for click-chemistry reaction and imaged using confocal microscopy. Protein synthesis is inhibited in both hair cells and supporting cells. Gentamicin induced inhibition of cellular protein synthesis shown to involve only hair cells (bottom panels). Scale bar 20 μm. **(D)** Immunoblot showing a decrease in cellular protein synthesis within organ of Corti explant lysates. AHA-biotin was detected with streptavidin (SA)-horseradish peroxidase (HRP). **(E)** Quantification of AHA uptake relative to immunoreactivity of MYO7A within mouse cochleae and utricles (*n* = 4). There is a marked dose-dependent reduction in AHA uptake in both sensory hair cells (HC) and supporting cells (SC). Error bars indicate SEM (standard error of the mean). **(F)** MYO7A immunoreactivity and nuclear morphology is not affected by short exposure (4 h) to high concentrations of cisplatin, demonstrating the appropriateness of using MYO7A staining to normalize the AHA signal.

For a relative quantification of AHA incorporation, the fluorescence intensity of the AHA-biotin-fluorophore conjugate was normalized to Myosin 7a (MYO7A) immunoreactivity, because it was consistent, strong and not affected by cisplatin exposure in the time frame relevant for AHA uptake measurements (4 h; see Figure 1F). For hair cells, the AHA signal was normalized to MYO7A of the same cell, and for supporting cells, AHA immunofluorescence was normalized to MYO7A of the adjacent hair cell. Staining procedures and confocal microscopy settings (gain, offset, laser power, magnification and z-stack numbers) were kept identical for all comparative experiments. For each experimental group, four organ of Corti were analyzed, with a minimum of 40 cells quantified per organ (mid frequency region).

### Immunoblots

**Figure 2:**
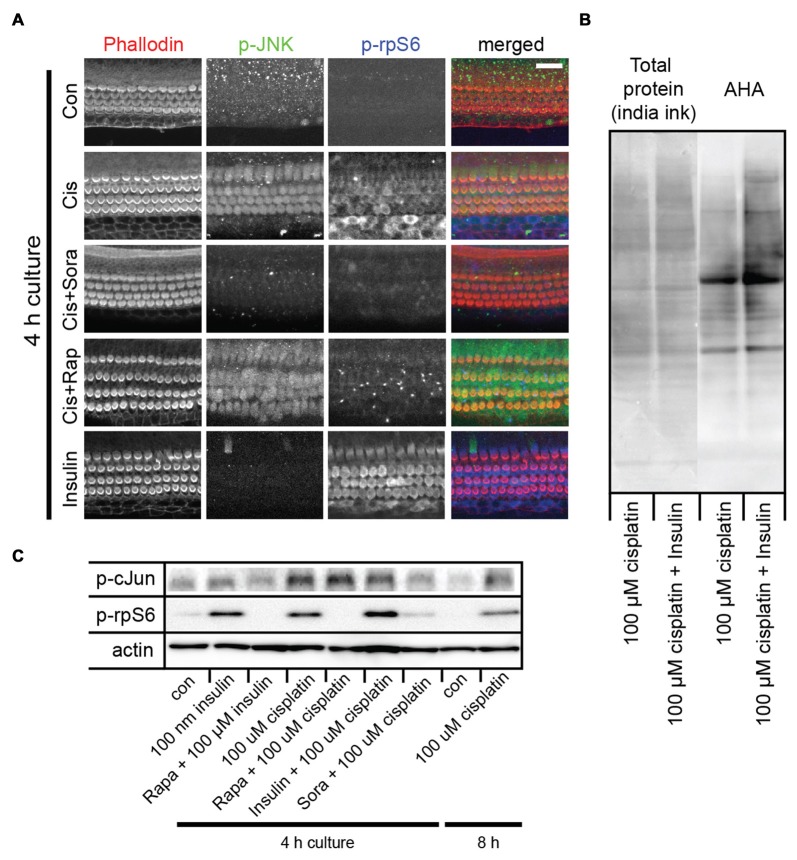
Cisplatin exposure activates both the c-Jun N-terminal kinase (JNK) and mammalian target of rapamycin (mTOR) pathways in sensory hair cells. **(A)** Cisplatin exposure resulted in a coordinated increase in phosphorylated JNK (p-JNK) and phosphorylated ribosomal protein S6 (p-rpS6) immunoreactivity, indicating an activation of the JNK and mTOR pathways. When explant cultures were exposed to both cisplatin and the multikinase inhibitor sorafenib (500 nM), the activation of JNK and mTOR pathways were inhibited. Rapamycin exposure did not alter the cisplatin-induced activation of JNK, but prevented activation of mTOR. When cultures were incubated with 100 nM insulin for 4 h, it resulted in robust activation of mTOR but not the JNK pathway (bottom panel). **(B)** AHA-biotin immunoblot showing that when administered with cisplatin, insulin lead to a 34% increase (*p*-value 0.004) in overall cellular protein synthesis, when compared to cisplatin alone. **(C)** Immunoblot confirming cisplatin-induced activation of the JNK pathway (as measured by p-c-Jun) and mTOR (as measured by p-rpS6), and the modulation of this response by sorafenib, rapamycin and insulin. Scale bar 20 μm.

Organs were homogenized in reducing SDS-PAGE sample buffer, heated to 70°C for 5 min, and microcentrifuged for 5 min to remove insoluble debris. Proteins were resolved using Bis-Tris SDS PAGE gel (Novex 4%–12%, Invitrogen, and TGX gels from Bio-Rad, CA, USA), transferred to PVDF membranes and stained with India Ink (total protein stain). Blots were then blocked in blocking buffer (ECL prime blocking reagent; GE Healthcare, UK) for 1 h and probed with the following primary antibodies overnight at 4°C: mouse anti phospho-JNK antibody (Thr183/Tyr185; catalog #9255, Cell Signaling, 1:1000), rabbit anti phospho-rpS6 antibody (Ser235/236; catalog #2211, Cell Signaling, 1:1000), rabbit anti-phospho-cJun (Ser73; cat #3270, 1:1000). After three 5 min washes in PBS/0.3% Tween 20, blots were incubated with HRP conjugated goat anti-rabbit secondary antibody (Cell Signaling Technology, Danvers, MA, USA) for 1 h, and bands were visualized by ECL reagent (Pierce Biotechnology, IL, USA; ECL Western blotting substrate and GE Healthcare GE ECL prime Western blotting reagent). Chemiluminescence was detected using an ImageQuant LAS4000 mini imager (GE Healthcare). The immunoblot for AHA incorporation (Figure 2B) was quantified by normalizing gray values from the AHA-biotin-SA-HRP signal to the gray value of corresponding india ink stain, which is a measure for total protein loading. Triplicate measurements were performed.

### Immunocytochemistry

Tissues were fixed for 25 min in 3% formaldehyde, washed three times for 5 min each in PBS, and incubated in blocking buffer (PBS containing 1% bovine serum albumin, 3% normal donkey serum and 0.2% saponin) for 1 h. Organs were then incubated with primary antibody overnight at room temperature in blocking buffer. Organs were washed three times for 5 min each with PBS and incubated with secondary antibodies (fluorophore-conjugated IgGs at 1:100; Invitrogen) and 0.25 μM phalloidin-Alexa 488 (Invitrogen) in the blocking solution for 1–3 h. Finally, organs were washed five times in PBS and mounted in Vectashield (Vector Laboratories, CA, USA). Samples were imaged using Zeiss LSM700 confocal microscopes. The following antibodies were used in this study: mouse anti-MYO7A antibody (Developmental Studies Hybridoma Bank, IA, USA, 1:100), mouse anti phosphorylated JNK (p-JNK) antibody (Thr183/Tyr185; catalog #9255, Cell Signaling, 1:100), rabbit anti phosphorylated ribosomal protein S6 (p-rpS6) antibody (Ser235/236; catalog #2211, Cell Signaling, 1:100), mouse anti gentamicin antibody (QED Biosciences, CA, USA, 1:100), rabbit anti cleaved caspase-3 antibody (Asp175; catalog #9661, Cell Signaling, 1:200).

### Hair Cell Counts

**Figure 3:**
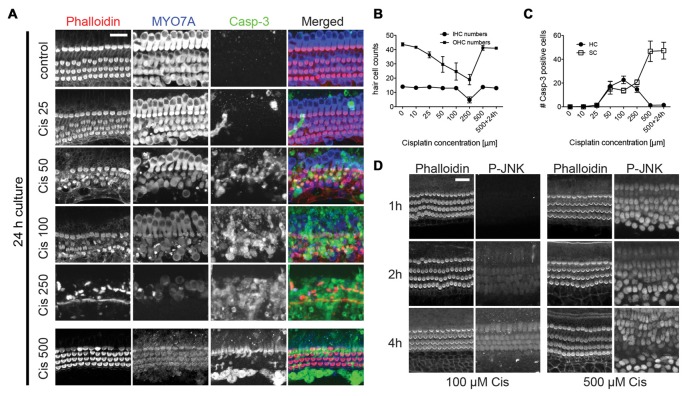
Cisplatin-induced hair cell death and dose-response curve. **(A–C)** Immunoreactivity of MYO7A and cleaved caspase-3 in organ of Corti explants with varying concentrations of cisplatin. Increasing cisplatin (cis) concentrations of up to 250 μM lead to an increase in hair cell death and caspase-3 positive hair cells. At very high concentrations (>500 μM) of cisplatin, however, there is a near complete protection of hair cells and an absence of cleaved caspase-3 staining in hair cells. Supporting cells exhibited strong caspase-3 immunoreactivity at higher concentrations. Outer hair cell (OHC) numbers were counted over a length of 100 μm in the basal turn. Caspase-3 positive cells were counted over a length of 200 μm of the basal turn. Error bars indicate SEM (standard error of the mean). *n* = 4 organs per experimental group. **(D)** Both moderate (100 μM) and high (500 μM) dose cisplatin activate JNK in hair cells (with different kinetics), suggesting that even at high doses, cisplatin uptake is not inhibited. Scale bar 20 μm.

**Figure 4:**
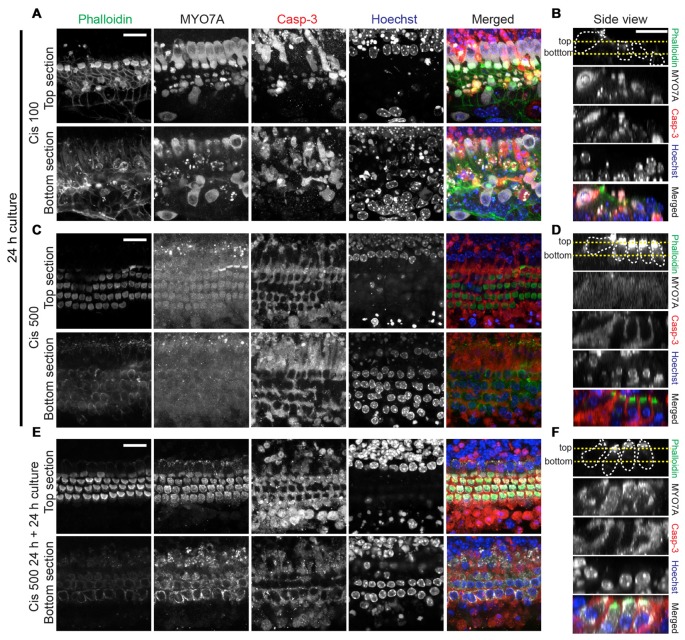
Analysis of hair cell integrity after exposure to high cisplatin concentrations. Explants were treated as follows: 24 h in 100 μM cisplatin **(A,B)**, 24 h in 500 μM cisplatin **(C,D)** or 24 h in 500 μM, followed by 24 h in normal growth medium **(E,F)**. Organs were then co-stained for F-actin (phalloidin, green), MYO7A (gray), cleaved casp-3 (red) and Hoechst (blue), colors are used in merged images only. In the merged images of **(C,D)**, the MYO7A (gray) signal, for which a high image gain was used for better visibility, was omitted. **(A,C,E)** are optical sections at the level of hair bundles (top section) and outer hair cell nuclei (bottom section). **(B,D,F)** are side views (generated by Reslice function in ImageJ), with the yellow dotted lines indicating the level of optical sections used in **(A,C,E)**. The protected hair cells at high cisplatin concentrations (500 μM) display normal nuclear morphology. Continuing the culture for 24 h in normal growth medium leads to near complete recovery of MYO7A immunoreactivity in hair cells initially exposed to 500 μM cisplatin. Scale bar 20 μm.

Hair cells were counted based on MYO7A or Phalloidin (in case MYO7A immunoreactivity was abolished despite presence of hair cell/bundle staining, as in Figures 3, 4) staining over a length of 100 μm of the basal turn of the cochlea, omitting the last 100 μm of the basal tip, which was often damaged during dissection. Activated caspase-3 immunoreactivity was counted over a stretch of 200 μm of the basal turn. For each experimental condition, at least four organ of Corti were analyzed. Exact numbers of organs (*n*) are indicated in the legends.

### Statistical Analysis

For statistical analysis, GraphPad Prism was used. One-way analysis of variance (ANOVA) was used to determine statistically significant differences between the means of the experimental groups. For pair-wise comparisons, a Tukey *post hoc* analysis was performed. *P* values smaller than 0.05 were considered significant. All *n* in statistical analyses refer to number of organs per experimental condition. All error bars indicate SEM.

## Results

### Cisplatin Inhibits Protein Synthesis in Organ of Corti Explants

We first sought to visualize and quantify, with cellular resolution, the effect of cisplatin on overall protein synthesis in organ of Corti explant cultures. This was achieved using the previously described BONCAT method (Dieterich et al., 2006), in which the incorporation of the methionine analog AHA into newly synthesized proteins serves as a proxy for overall protein synthesis activity. Figure 1A illustrates the molecular structure of AHA as it compares to methionine. Figure 1B illustrates the BONCAT technique using either cell lysates for immunoblot or fixed organs for fluorescence microscopy. Organ of Corti explants from 3 to 4 day old mice were cultured in growth medium containing AHA, in the presence of varying cisplatin concentrations. After 4 h, prior to onset of cisplatin-induced cell death, explants were fixed and processed for click-chemistry reaction and imaged using confocal microscopy. As evident in Figure 1C, cisplatin inhibits AHA incorporation, thus protein synthesis, in a concentration-dependent manner (quantified in Figure 1E). Cisplatin inhibited protein synthesis in all cell types in the organ of Corti, including hair cells and supporting cells. This is in contrast to the pattern of protein synthesis inhibition elicited by aminoglycosides, which is restricted to hair cells (Figure 1C, bottom panels). As shown in Figure 1F, cochlear hair cells display normal nuclear morphology and MYO7A immunoreactivity after 4 h of culture, even at very high cisplatin concentrations (750 μM), demonstrating the appropriateness of using MYO7A levels for normalizing the AHA signal. A similar effect of cisplatin on protein synthesis was seen in utricle explants (no images shown, quantification in Figure 1E, bottom). The reduction of protein synthesis was also evident in immunoblot experiments of organ of Corti explant lysates, in which AHA-biotin was detected with SA-HRP (Figure 1D). In summary, we demonstrated that cisplatin inhibits protein synthesis in a dose-dependent manner in all cell types under organ of Corti explant culture conditions, including hair cells and surrounding supporting cells.

### Cisplatin Activates JNK and mTOR Pathways, While Insulin Activates mTOR and Stimulates Cellular Protein Synthesis

We previously demonstrated that aminoglycoside antibiotics activate the JNK and mTOR pathways (Francis et al., 2013). This activation was also noted to have correlated with the inhibition of cellular protein synthesis. The activation of the mTOR pathway was proposed to be a compensatory response to protein synthesis inhibition (Francis et al., 2013). To test whether cisplatin resulted in a similar stress response, mouse organ of Corti explant cultures were exposed to 100 μM cisplatin, and JNK and mTOR activation was detected by p-JNK and p-rpS6 immunoreactivity, respectively. As was found with gentamicin, cisplatin exposure resulted in a coordinated increase in p-JNK and p-rpS6 immunoreactivity, indicating an activation of the JNK and mTOR pathways (Figure 2A). We next tested whether activation of JNK and mTOR is modulated by pharmaceutical compounds. Sorafenib is an FDA-approved drug used as an adjunct in chemotherapeutic strategies for renal cell, hepatocellular and thyroid carcinomas (Blair and Plosker, 2015; Gadaleta-Caldarola et al., 2015). It is a multikinase inhibitor and is known to inhibit VEGFR, PDGFR as well as the MAP3K and MLK7. MLK7 (aka ZAK) has been shown to be activated in apoptosis associated with the ribotoxic stress response (Wang et al., 2005; Jandhyala et al., 2008; Sauter et al., 2010). We have previously demonstrated that aminoglycosides elicit a similar ribotoxic stress response within sensory hair cells and that sorafenib inhibits JNK activation. Sorafenib was also found to confer a partial protection from gentamicin-induced hair cell death (Francis et al., 2013). To determine if cisplatin-induced JNK activation can be prevented by sorafenib, we incubated mouse cochlea cultures in 500 nM sorafenib for 1 h, prior to incubation in 100 μM cisplatin. Strikingly, sorafenib nearly completely prevented cisplatin-induced JNK phosphorylation (Figure 2A). In addition to the prevention of JNK activation, sorafenib also inhibited the phosphorylation of rpS6 (Figure 2A). We then tested whether the prototypical mTOR inhibitor, rapamycin, inhibits JNK and/or mTOR activation. As expected, rapamycin inhibited the phosphorylation of rpS6 (Figure 2A). However, unlike sorafenib, rapamycin did not alter cisplatin-induced activation of the JNK pathway (Figure 2A), indicating JNK activation occurs upstream of mTOR. Cisplatin-induced activation of the JNK pathway (as measured by p-c-Jun) and mTOR (as measured by p-rpS6), and the modulation of this response by sorafenib, rapamycin and insulin, were validated in immunoblot experiments (Figure 2C).

Next, we sought a way to counteract cisplatin-induced protein synthesis inhibition using pharmacological means. It is well established that insulin activates mTOR and cellular protein synthesis (Proud, 2006). Indeed, when cultures were incubated with 100 nM insulin for 4 h, we observed a robust activation of the mTOR pathway (Figure 2A, bottom). Insulin did not, however, activate the JNK pathway (Figure 2A, bottom). At the same time, insulin induced an increase in overall cellular protein synthesis, as demonstrated in an AHA-biotin immunoblot experiment (Figure 2B; 34% increase of AHA-biotin signal in cultures treated with cisplatin and insulin, compared to cultures treated with cisplatin alone). In summary, we showed that cisplatin activates both the JNK and the mTOR pathways. Inhibition of JNK by sorafenib prevents both JNK and mTOR activation, while rapamycin only prevents mTOR activation, demonstrating that mTOR is downstream of JNK in this particular stress pathway. Furthermore, we show that insulin activates mTOR and stimulates protein synthesis, independent of the JNK pathway.

### Cisplatin-Induced Hair Cell Death and Dose-Response Curve

Next, we analyzed hair cell death in organ of Corti explants exposed to cisplatin. Explant cultures were exposed to concentrations of cisplatin ranging from 10 μM to 500 μM, over 24 h. Hair cells were counted using MYO7A and phalloidin reactivity. Apoptotic events were visualized using cleaved caspase-3 immunoreactivity. We observed a surprising dose-response relationship (Figures 3A–C): increasing cisplatin concentrations of up to 250 μM lead to an increase in hair cell death and caspase-3 positive hair cells. At very high concentrations (>500 μM) of cisplatin, however, we observed a near complete inhibition of hair cell loss and absence of cleaved caspase-3 staining in hair cells, while supporting cells exhibited strong caspase-3 immunoreactivity. This pattern of seemingly paradoxical hair cell protection with very high doses of cisplatin is similar to a previously reported finding (Ding et al., 2011). Next, we sought to demonstrate that rescue of hair cells at very high concentrations is not caused by a reduced uptake of cisplatin into cochlear cells. As demonstrated in Figure 2, cisplatin causes a robust, dose-dependent activation of the JNK pathway, as visualized by detection of p-JNK. We employed this phenomenon as a surrogate marker for cisplatin entry into cells. We examined the time-dependent activation of JNK at moderate cisplatin concentration, as well as very high cisplatin concentrations. At lower cisplatin concentrations (100 μM), JNK is activated preferentially in hair cells, increasing over a time period of 2–4 h. At 500 μM, however, JNK immunoreactivity can be found broadly in hair cells and supporting cells after only 1 h, remaining at high levels up to 4 h (Figure 3D). This is consistent with a model in which cisplatin uptake increases with its dose, and hair cells more readily take up cisplatin compared to supporting cells. Our data thus suggests that uptake of cisplatin into organ of Corti cells is likely not inhibited at very high cisplatin concentrations.

Next, we further investigated the nature of the hair cells surviving at high cisplatin concentrations, by correlating MYO7A and caspase-3 immunoreactivity with appearance of nuclei. Significant hair cell loss is observed at moderate cisplatin concentrations (100 μM), accompanied by cleaved caspase-3 immunoreactivity and nuclear fragmentation in hair cells (Figures 4A,B). At very high cisplatin concentrations, however, hair cells are free of caspase-3 immunoreactivity, and the hair cell nuclei display normal morphology, while supporting cells exhibit strong caspase-3 immunoreactivity and fragmented nuclei (Figures 4C,D). Moreover, when the culture was continued in normal growth medium for another 24 h (without cisplatin), the hair cells remained seemingly healthy, free of caspase-3 and of normal nuclear appearance (Figures 4E,F, quantified in Figures 3B,C).

Several other observations are worth noting. Hair cell death was preceded by a weakening of MYO7A immunoreactivity, starting with outer hair cells, and at higher cisplatin concentrations, also affecting the inner hair cells (Figure 3A). This suggests that MYO7A level in hair cells is affected by the protein synthesis inhibition. At very high cisplatin concentrations (500 μM, 24 h culture), the MYO7A immunoreactivity has weakened to a degree that hair cells are barely detectably based on MYO7A staining (Figures 4C,D). However, hair cell bodies are clearly present, as evident in the phalloidin and MYO7A signal (at higher image gain) and the presence of nuclei (Hoechst staining; Figures 4C,D). Interestingly, these hair cells nearly fully recovered their MYO7A immunoreactivity when cultured without cisplatin for another 24 h (Figures 4E,F), suggesting that these hair cells retain a survival benefit. In summary, we report that moderate cisplatin concentrations cause hair cell death and removal (possibly by supporting cells), but that very high concentrations of cisplatin abolishes this effect, preventing hair cell death and removal.

### High-Dose Cisplatin Cross-Protects Against Gentamicin Ototoxicity in Sensory Hair Cells

**Figure 5:**
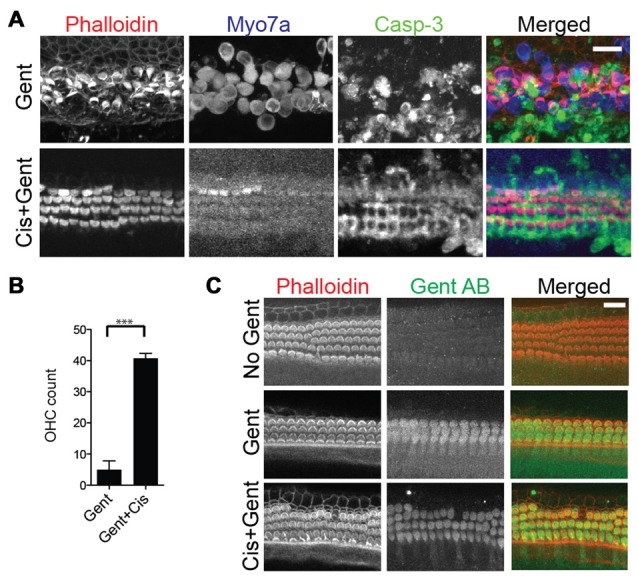
High-dose cisplatin cross-preserves hair cells exposed to toxic doses of gentamicin. **(A)** Mouse organ of Corti explant cultures exposed to 100 μM gentamicin (Gent) for 24 h results in significant hair cell loss (top panels). Additional supplementation of 500 μM cisplatin to the toxic concentration of gentamicin rescues the hair cells, albeit with severely reduced MYO7A immunoreactivity (bottom panels). **(B)** Outer hair cell counts per 100 μm (basal turn) after exposure to gentamicin with or without high dose cisplatin (Unpaired *t*-test, *p*-value: < 0.0001, *n* = 6 organs). **(C)** Gentamicin uptake into cochlear cells was visualized by immunocytochemistry using a gentamicin-specific antibody. There was no difference in gentamicin immunoreactivity within the cochlear hair cells between gentamicin alone or with high dose cisplatin. Scale bar 20 μm.

What would cause the rescue of hair cells at very high concentrations of cisplatin? Previous studies have shown that high concentrations of protein synthesis inhibitors such as cycloheximide can prevent hair cell death (Matsui et al., 2002), possibly due to the fact that regulated forms of cell death themselves depend on proteinaceous factors, such that complete shutdown of protein synthesis even prevents apoptotic cell death. If this was true, we reasoned that high concentrations of cisplatin should also prevent the cell death and removal of hair cells caused by other ototoxins such as aminoglycosides. This was indeed the case; exposure to 100 μM gentamicin for 24 h causes significant hair cell loss, while additional supplementation of 500 μM cisplatin rescues the hair cells, albeit with severely reduced MYO7A immunoreactivity (Figures 5A,B). One potential explanation for this apparent broad hair cell protection was the possibility that gentamicin uptake into hair cells is inhibited by cisplatin. To test this, gentamicin taken up into cochlear cells was detected using a gentamicin-specific antibody. There was no difference in gentamicin immunoreactivity within the cochlear hair cells between gentamicin alone or with high dose cisplatin (Figure 5C). This indicates that, although high dose cisplatin confers a protective effect from gentamicin toxicity, this is not a result of prevention of gentamicin uptake. We suggest that the protective effect of high cisplatin concentrations is directly caused by a complete shutdown of protein synthesis (discussed later).

### Preventing JNK Activation and Stimulating Protein Synthesis Ameliorates Cisplatin-Induced Hair Cell Death

**Figure 6:**
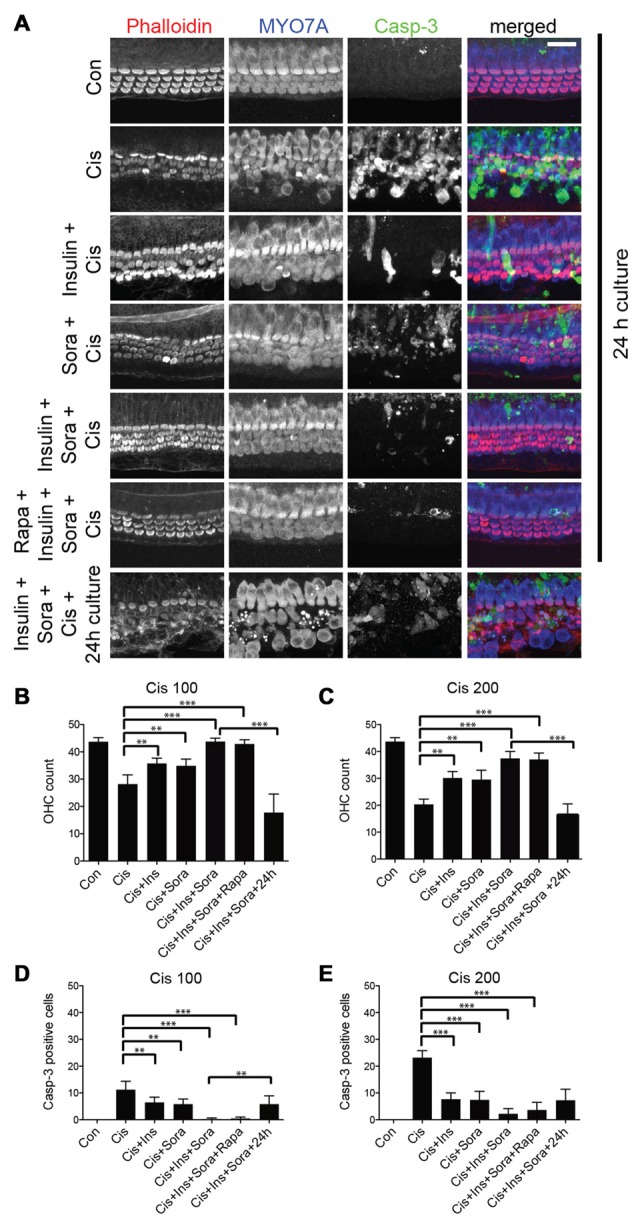
Inhibition of JNK activation with simultaneous stimulation of protein synthesis results in protection from cisplatin ototoxicity. **(A)** Cisplatin exposure (100 μM) results in significant loss of outer hair cells (second row). Co-incubation of cisplatin with either insulin or sorafenib results in a small, but significant protection of outer hair cells (3rd, 4th rows). Combined incubation with cisplatin, insulin and sorafenib provided strong protection of cochlear hair cells (5th row). This protection, however, did not persist when organs were cultured for another 24 h in normal growth medium (7th row). Addition of rapamycin, a known inhibitor of the mTOR pathway, did not reverse the protection seen with sorafenib and insulin (6th row). **(B)** Quantification of outer hair cell numbers demonstrating the protective effect of insulin (Ins), sorafenib (Sora) and the combination of the two after 100 μM cisplatin (Cis) exposure. *P*-values for analysis of variance (ANOVA) with *post hoc* test (Tukey): Con to Cis: < 0.0001, Cis to Cis+Ins and Cis+Sora: < 0.001, Cis to Cis+Ins+Sora: < 0.0001. **(C)** Same as **(B)**, but after 200 μM cisplatin exposure. *P*-values for ANOVA with *post hoc* test (Tukey): Con to Cis: < 0.0001, Cis to Cis+Ins and Cis+Sora: < 0.001, Cis to Cis+Ins+Sora: < 0.0001. **(D)** Quantification of cleaved Caspase-3 (Casp-3)-positive cells, demonstrating the protective effect of insulin (Ins), sorafenib (Sora) and the combination of the two after 100 μM cisplatin (Cis) exposure. *P*-values for ANOVA with *post hoc* test (Tukey): Con to Cis: < 0.001, Cis to Cis+Ins and Cis+Sora: < 0.001, Cis to Cis+Ins+Sora: < 0.0001.** (E)** Same as **(D)**, but after 200 μM cisplatin exposure. *P*-values for ANOVA with *post hoc* test (Tukey): Con to Cis: < 0.0001, Cis to Cis+Ins and Cis+Sora: < 0.0001, Cis to Cis+Ins+Sora: < 0.0001. Asterisks in plots indicate *p*-values (***< 0.0001, **< 0.001). To reduce unnecessary complexity in the plots, only a subset of the *p*-values of the pair-wise *post hoc* tests are displayed. Inner hair cells were not affected in all experimental groups (not included in quantification). Scale bar 20 μm.

Next, we explored whether drug-mediated modulation of the JNK and/or mTOR pathway, in conjunction with a stimulation of cellular protein synthesis, could be used to alleviate cisplatin ototoxicity in culture. We have previously shown that while sorafenib inhibits gentamicin-induced activation of JNK, it provides only partial protection from hair cell loss (Francis et al., 2013). This suggests that there are other pathways contributing to cellular apoptosis in addition to JNK. We sought to determine whether combinatorial prevention of JNK activation (using sorafenib), inhibition of mTOR (using rapamycin) and stimulation of overall protein synthesis (using insulin) might result in the protection from cisplatin-induced hair cell death. As expected, exposure to cisplatin caused significant hair cell death (Figure 6A). When the cisplatin-exposed organs were co-cultured with either insulin or sorafenib, there was a small increase in the number of surviving outer hair cells. Simultaneous application of both sorafenib and insulin, however, lead to a significant rescue of cisplatin-exposed hair cells (Figure 6A, 5th row). This preservation also correlated with the number of caspase-3 positive cells (Figure 6C). To determine if the added hair cell protection conferred by insulin was related to activation of the mTOR pathway, cisplatin-exposed organ of Corti were co-cultured with sorafenib, insulin and rapamycin. The addition of rapamycin did not alter the hair cell preservation seen with sorafenib and insulin (Figures 6A,B). This suggests that the combined protective effects of sorafenib and insulin are independent of the mTOR pathway. Finally, we tested whether the protective effect of insulin and sorafenib persisted when removed from the culture medium. Unlike the continued protection of hair cells after exposure to very high cisplatin concentrations (>500 μM; Figures 3, 4), the protection conveyed by combined application of insulin and sorafenib was not sustained when the cultures were continued in normal growth medium (Figure 6A, bottom row, and Figures 6B–E). In summary, we demonstrate that cisplatin-induced ototoxicity in culture is alleviated by simultaneous inhibition of JNK activation and stimulation of protein synthesis. Both manipulations have an additive, otoprotective effect, suggesting the respective pathways operate independently.

### Pre- and Post-Treatment with Sorafenib and Insulin Provide Equal Hair Cell Protection

**Figure 7:**
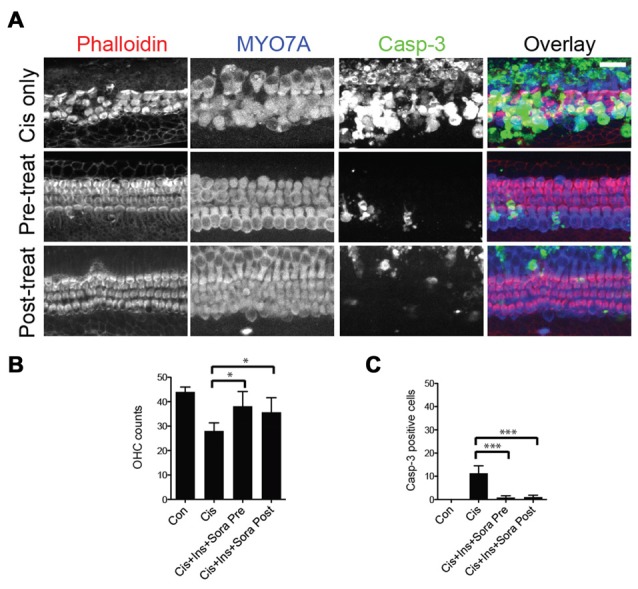
Effects of timing of treatment with sorafenib and insulin on hair cell protection from cisplatin-induced cell death. **(A–C)** Cisplatin exposure results in marked immunoreactivity of caspase-3 and loss of outer hair cells. There is significant protection of outer hair cells and limited immunoreactivity of caspase-3 with either pre- (1 h treatment prior to 200 μM cisplatin) or post-treatment (added after 1 h exposure to cisplatin) with 500 nM sorafenib and 100 nM insulin. Differences between pre- and post-treatment paradigms were not significant, though each were significantly different from cisplatin alone. ANOVA with *post hoc* test (Tukey) was performed for statistical analysis. Asterisks in plots indicate *p*-values (***< 0.0001, *< 0.01). Inner hair cells were not affected in all experimental groups (not included in quantification). Scale bar 20 μm.

Finally, to determine whether the timing of treatment with sorafenib and insulin relative to cisplatin exposure altered their protective effects, we compared pre- and post-treatment paradigms. In the former, organ of Corti explants were cultured with sorafenib and insulin 1 h prior to being incubated with cisplatin. In the post-treatment group, the organs were incubated first with cisplatin, followed 1 h later with sorafenib and insulin. Both pre- and post-treatment with sorafenib and insulin resulted in a similar degree of hair cell protection, indicating both paradigms are effective in inhibiting cisplatin-induced hair cell death (Figures 7A–C).

## Discussion

Despite the lack of similarity in molecular structure, aminoglycosides and cisplatin exhibit significant overlap in the stress response they elicit in sensory hair cells: to mention a few, both elicit oxidative stress (Lautermann et al., 1995; Clerici et al., 1996; Hirose et al., 1997; Kopke et al., 1997; Dehne et al., 2000), activate p53 (Zhang et al., 2003; Coffin et al., 2013; Benkafadar et al., 2017) and the JNK pathway (Wang et al., 2004; Francis et al., 2013). A comprehensive summary for the commonalities and differences in aminoglycoside and cisplatin ototoxicity is presented in the review by Schacht et al. (2012). We have previously demonstrated that aminoglycosides cause a stress response reminiscent of ribotoxic stress, involving activation of the JNK and mTOR pathways and inhibition of cellular protein synthesis (Francis et al., 2013). In this study, we demonstrate that, like aminoglycosides, cisplatin causes a significant reduction in cellular protein synthesis within sensory hair cells in culture. In contrast to aminoglycosides, cisplatin inhibits protein synthesis in all cochlear cell types. We suggest this is due to differences in uptake specificity. Aminoglycosides are highly preferentially taken up by hair cells through the mechanotransduction channel (Marcotti et al., 2005; Waguespack and Ricci, 2005; Wang and Steyger, 2009; Alharazneh et al., 2011), while cisplatin might enter cells through a more generic pathway (Sinani et al., 2007; Ding et al., 2011; Ciarimboli, 2014). It should be noted, however, that cisplatin also displays a slight preference for hair cells compared to supporting cells: while high concentrations of cisplatin (500 μM) activate JNK in hair cells and supporting cells, lower doses of cisplatin (100 μM) activate JNK in hair cells only (Figure 3D), This is consistent with a previous report suggesting that cisplatin also enters the hair cell through the mechanotransduction channel (Thomas et al., 2013). Nevertheless, we suggest that compared to aminoglycosides, cisplatin affects various cell types in a broader manner. We believe that the difference in cellular damage profile is highly relevant for developing therapeutic strategies, in that prevention of aminoglycoside ototoxicity might be primarily directed to hair cells, while addressing cisplatin ototoxicity should require a broader protective effort.

Cisplatin’s effect on blocking protein synthesis might also offer an explanation for a curious dose-response curve, first reported by the Salvi lab (Ding et al., 2011), and also confirmed in our present study. Cisplatin caused an increasing loss of hair cells up to a concentration of 250 μM. At very high concentrations of cisplatin, however, there appeared to be a protective effect on sensory hair cells. Ding et al. (2011) postulated that this may be caused by the complex interplay of various copper transporters, which enable cisplatin uptake into hair cells. We suggest that additional mehanisms could contribute to this phenomenon; at moderate concentrations (below 250 μM), cisplatin causes the expected stress and cell death response. At concentrations above that level, hair cells experience a complete arrest of protein synthesis, inhibiting all cellular processes, including cell death programs. The strongest indication for a general arrest of cell death programs is presented in our observation that very high concentrations of cisplatin cross-protects against aminoglycoside ototoxicity, suggesting this protection is not specific to cisplatin.

What is the nature of the hair cells surviving at high cisplatin concentrations? One possibility is that the supposedly rescued hair cells are in fact committed to cell death, but fail to initiate or complete the cell death program. These hair cells would be retained in the epithelium, since supporting cells at such high cisplatin levels undergo apoptosis (as evident in strong cleaved caspase-3 immunoreactivity in Figures 3A, 4C–F) and might loose their ability to phagocytose dying hair cell (Monzack et al., 2015). Experiments detailed in Figure 4, however, demonstrate that the hair cells exposed to high cisplatin doses remain healthy after cisplatin is washed out, at least for 24 h in continued culture. We therefore have to assume that these hair cells sustain a survival benefit in absence of high cisplatin doses. The significance and therapeutic usefulness of this protection is unclear. First, such high cisplatin concentrations (>500 μM) are irrelevant for clinical considerations, with typical plasma concentrations ranging between 600 nM and 20 μM (Urien et al., 2005). Possibly more detrimental, such high cisplatin concentrations induce apoptosis in supporting cells, which will inevitably affect the function and survival of the sensory epithelium. Neverthelss, learning the basis of this curious protection will provide important clues for understanding basic mechanisms of cisplatin ototoxicity.

What is the mechanism by which cisplatin inhibits protein synthesis? We have previously shown that the usual pathways affecting protein synthesis, most notably stress responses like the unfolded protein response or an inhibition of the mTOR pathway, are not causative with aminoglycoside-induced protein synthesis inhibition (Francis et al., 2013). Instead, we provided evidence that aminoglycosides directly bind and inhibit ribosomal RNA (Scheunemann et al., 2010; Francis et al., 2013). Such binding activity, negligible in most other cell types, reaches toxic levels in hair cells, which possess the unfortunate property of accumulating aminoglycosides. Despite differences in molecular structure between aminoglycoside antibiotics and cisplatin, there is some evidence that also cisplatin might bind to RNA; Heminger et al. (1997) demonstrated that cisplatin crosslinks mRNA and rRNA, causing translational arrest in reticulocytes. And just recently, the crystal structure of cisplatin bound to RNA was reported (Melnikov et al., 2016). It is therefore conceivable that cisplatin elicits a ribotoxic stress response similar to toxins such as ricin, and as we proposed in our previous study, aminoglycoside antibiotics. Cisplatin could, however, also affect protein synthesis through its canonical DNA-crosslinking activity, accepted to be the main mechanism underlying its cytotoxic effect (Eastman, 1987). DNA crosslinking causes genotoxicity, which is a well-established cause for protein synthesis inhibition (Sheikh and Fornace, 1999; Braunstein et al., 2009). In summary, we believe that cisplatin behaves like a “dirty bomb”, affecting protein synthesis through various ways, but also eliciting other stress signaling such as the JNK pathway; all of which contribute to the overall ototoxicity. While such multi-faceted toxicity is a boon for killing cancer cells, it represents a great challenge when it comes to preventing toxic side effects, necessitating a multi-pronged approach. Our present study points to several potential avenues for intervention, all of which might have to be targeted simultaneously.

### JNK Pathway

It is well documented that various ototoxic stressors, including cisplatin, result in activation the JNK pathway (Zine and Romand, 1996; Pirvola et al., 2000; Matsui et al., 2004). Despite a proposed role of JNK activation in initiating apoptosis, whether JNK is necessary for, or causal of, apoptosis is controversial (Liu and Lin, 2005). In the case of cisplatin ototoxicity, a previous study has shown that inhibition of the JNK pathway does not result in protection of auditory hair cells (Wang et al., 2004). Our results confirmed that near complete blockade of JNK with sorafenib does not provide an equal benefit for hair cell survival. However, our study suggests that JNK inhibition can synergize with other protective measures.

### Protein Synthesis

If protein synthesis inhibition is indeed a significant contributor to ototoxicity, how does this knowledge guide the development of preventative or ameliorative strategies? Our success using insulin, a general stimulating agent of protein synthesis, points in one potentially beneficial direction. Future strategies must be based on the understanding that protein synthesis inhibition, like so many other stress responses, is a double-edged sword, and that finding the right balance is the key. Blocking the synthesis of new proteins will most affect proteins with high turnover (Adams and Cooper, 2007). Therefore, to tip the balance of pro-survival and pro-death factors to the former, it is crucial to understand the overall proteostasis (sum of synthesis and degradation) of cell stress and death factors, with expected differences in different cell types.

Protein synthesis is regulated on so many levels, and affects so many aspects of cellular signaling that its targeted and isolated manipulation is intractable. The otoprotective effect of insulin, for example, might be mediated by mechanisms independent from protein synthesis stimulation. Several previous reports have described that growth factors such as IGF-1, EGF, TGF alpha and insulin protect against various ototoxic insults (Romand and Chardin, 1999; Iwai et al., 2006; Lou et al., 2015; Yamahara et al., 2015). In the case of IGF-1, protection was mediated by supporting cells (Yamahara et al., 2015). Insulin also activates phosphatidylinositol-4,5-bisphosphate 3-kinase (PI3K)- protein kinase B (Akt) signaling, which has been shown to be protective against both aminoglycoside and cisplatin ototoxicity, potentially through activation of pro-survival MAPK signaling and/or inactivation or downregulation of pro-apoptotic proteins (Lizcano and Alessi, 2002; Chung et al., 2006; Brand et al., 2011; Jadali and Kwan, 2016; Jadali et al., 2017). In addition, insulin-activated Akt is known to inhibit glycogen synthase kinases (GSKs), and inhibition of GSK-3 activity has been shown to inhibit cisplatin ototoxicity in auditory cells (Park et al., 2009; Kim et al., 2014; Hermida et al., 2017). Alternatively, insulin also inhibits AMP-activated protein kinase (AMPK) activity (Towler and Hardie, 2007). AMPK has been shown to play a role in noise-induced hearing loss, and inhibition of AMPK activation protects hair cells and ribbon synapses against acoustical overstimulation (Nagashima et al., 2011; Zheng et al., 2014; Hill et al., 2016). Further studies are required to obtain a full understanding of the mechanisms by which insulin confers otoprotection against cisplatin, and how much of this protection is attributable to an increase in protein synthesis. It should be noted, however, that the mTOR stimulating effect of insulin does not seem to play a role in the protective effect, since mTOR inhibition by rapamycin did not reverse insulin-mediated hair cell protection (Figure 6).

Countless other pathways and corresponding protective interventions have been reported and reviewed extensively (Rybak et al., 2008, 2007; Schacht et al., 2012; Waissbluth and Daniel, 2013; Karasawa and Steyger, 2015), underscoring the notion that no single “magic bullet” will achieve the clinically relevant level of protection, and that a combination of interventions, accompanied by a thorough understanding of the underlying mechanisms, must be sought.

## Conclusion

We found that cisplatin inhibits cellular protein synthesis in organ of Corti explant cultures. Similar to gentamicin, cisplatin also activates the JNK. Simultaneous stimulation of cellular protein synthesis by insulin, and inhibition of JNK activation by sorafenib, significantly improved hair cell survival in culture. The presented data thus suggest that protein synthesis inhibition might be a potential contributor to cisplatin-mediated ototoxicity.

## Author Contributions

This study was designed by BDN, SF and J-BS. Experiments were performed by BDN, SF, ELW, SZ and J-BS; data was analyzed by BDN and J-BS; this article was written by BDN, SF and J-BS.

## Conflict of Interest Statement

The authors declare that the research was conducted in the absence of any commercial or financial relationships that could be construed as a potential conflict of interest.
